# Data concerning the fractionation of individual whey proteins and casein micelles by microfiltration with ceramic gradient membranes

**DOI:** 10.1016/j.dib.2019.104102

**Published:** 2019-06-05

**Authors:** Hans-Jürgen Heidebrecht, Ulrich Kulozik

**Affiliations:** a Chair of Food and Bioprocess Engineering, Technical University of Munich, Germany; b ZIEL Institute for Food & Health, Technical University of Munich, Germany

**Keywords:** Immunoglobulins, Microfiltration, IgG

## Abstract

Data are related to the research article “Fractionation of casein micelles and immunoglobulins by microfiltration in diafiltration mode Study of the transmission and yield of IgG, IgA and IgM” [1]. The data show the transmission and yield of the individual whey proteins α-Lactalbumin (α-La), β -Lactoglobulin (β -Lg), blood serum albumin (BSA), lactoferrin (LF), lactoperoxidase (LPO) and the immunoglobulins IgG, IgA, IgM during microfiltration (0.14 μm) performed in diafiltration mode at 50 °C with different applied transmembrane pressures (0.6-3 bar). The data provide information on the decrease of the respective proteins in the microfiltration retentate and their increase in the UF retentate. The relevant analytical methods for the individual protein detection were performed by reversed phase high performance liquid chromatography and ELISA. The isoelectric point of IgG and IgM was measured with the Zetasizer Nano ZS.

**Table:** Specifications Table

Subject area	*Chemistry, biology*
More specific subject area	*Fractionation of individual whey proteins by microfiltration*
Type of data	*Graphs, figure*
How data was acquired	Microfiltration pilot plant, *Zetasizer Nano ZS, SDS-PAGE, reversed phase high performance liquid chromatography*
Data format	*Analyzed*
Experimental factors	*Fat (centrifugation), casein (microfiltration), lactose/minerals (ultrafiltration) removed from raw colostrum or milk to obtain whey*
Experimental features	*Determination of individual whey proteins during microfiltration at different process conditions*
Data source location	Technical University of Munich *(Freising), Germany*
Data accessibility	*With this article*
Related research article	*Data is provided as additional material directly related to the article H.-J. Heidebrecht, U. Kulozik, Fractionation of casein micelles and minor proteins by microfiltration in diafiltration mode: Study of the transmission and yield of the immunoglobulins IgG, IgA and IgM, Int Dairy J, 93 (in press), 2019, 1–10*[1]*.*

**Table:** 

**Value of the data** • Transmission yield of the individual whey protein at different TMP • Data deliver information on how to operate the microfiltration process during the fractionation of casein micelles and whey proteins with the assessment criteria time and yield • Data are valuable for the design of filtration plants with the aim of recovering the individual whey protein fractions in the microfiltration permeate

## 1 Data

The dataset contains information on the transmission and yield of individual whey proteins during milk protein fractionation by microfiltration. Furthermore, data on the comparison of analytical methods for the determination of bovine IgG and β-Lg are as well as the influence of temperature during filtration on the respective whey proteins are presented.

**Fig. 1:**
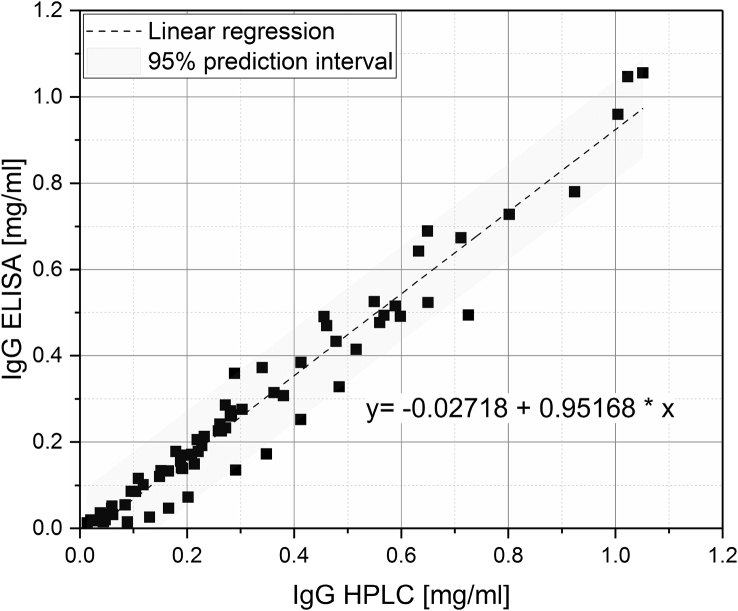
Linear correlation of the IgG concentration measured with RP-HPLC and ELISA.

**Fig. 2:**
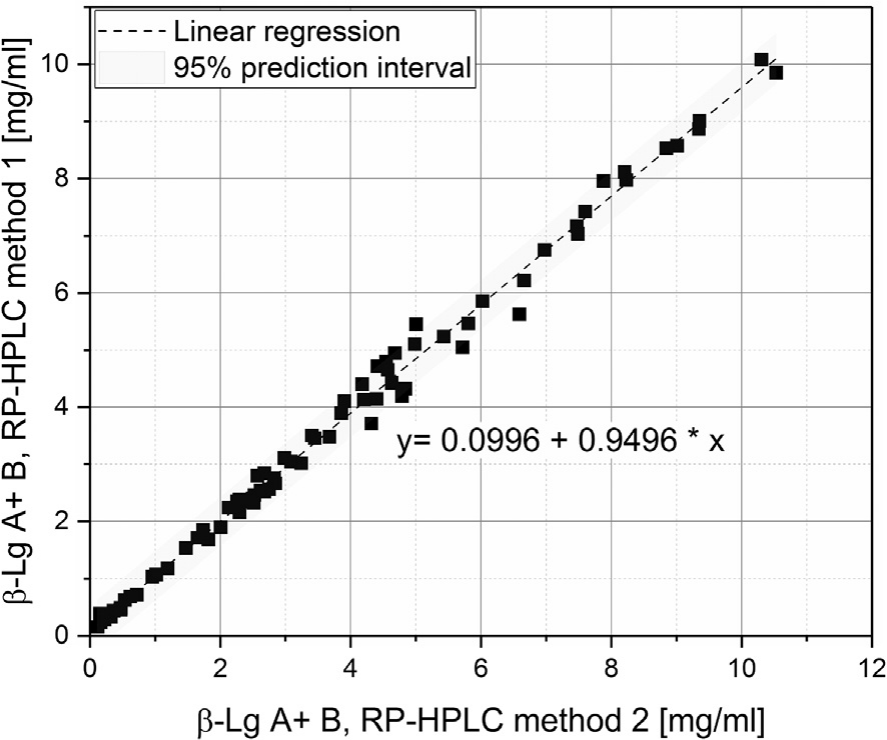
Linear correlation of β-Lg measured with two independent RP-HPLC methods (B); method 1 according to Ref. [6], method 2 according to Ref. [7].

**Fig. 3:**
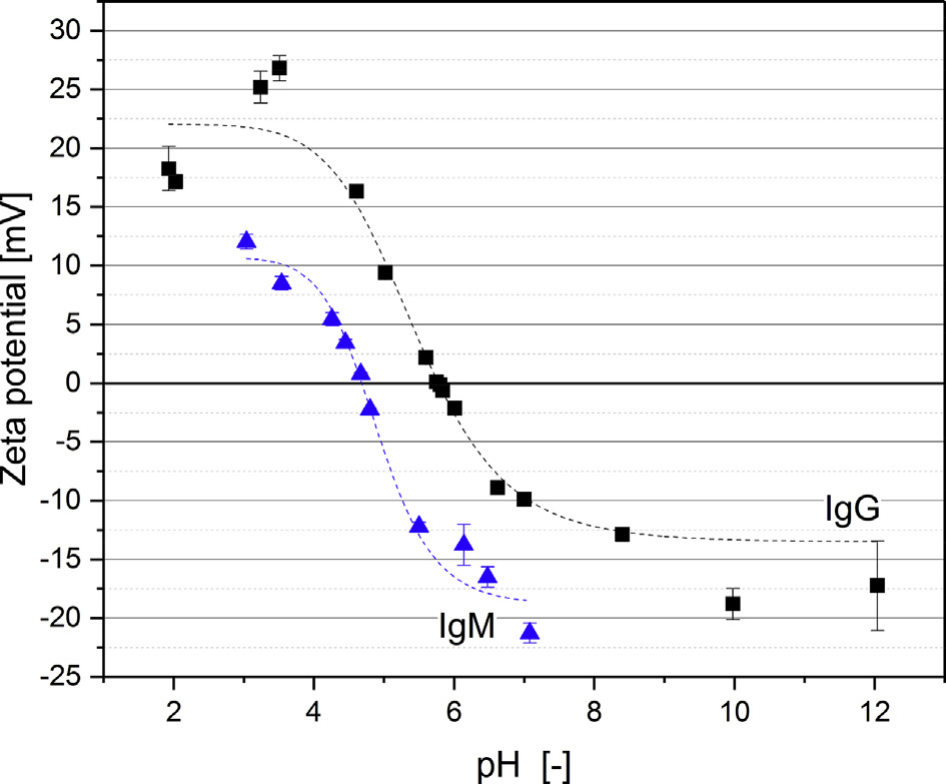
Zeta potential as function of the pH from isolated IgG and IgM for IEP determination.

**Fig. 4:**
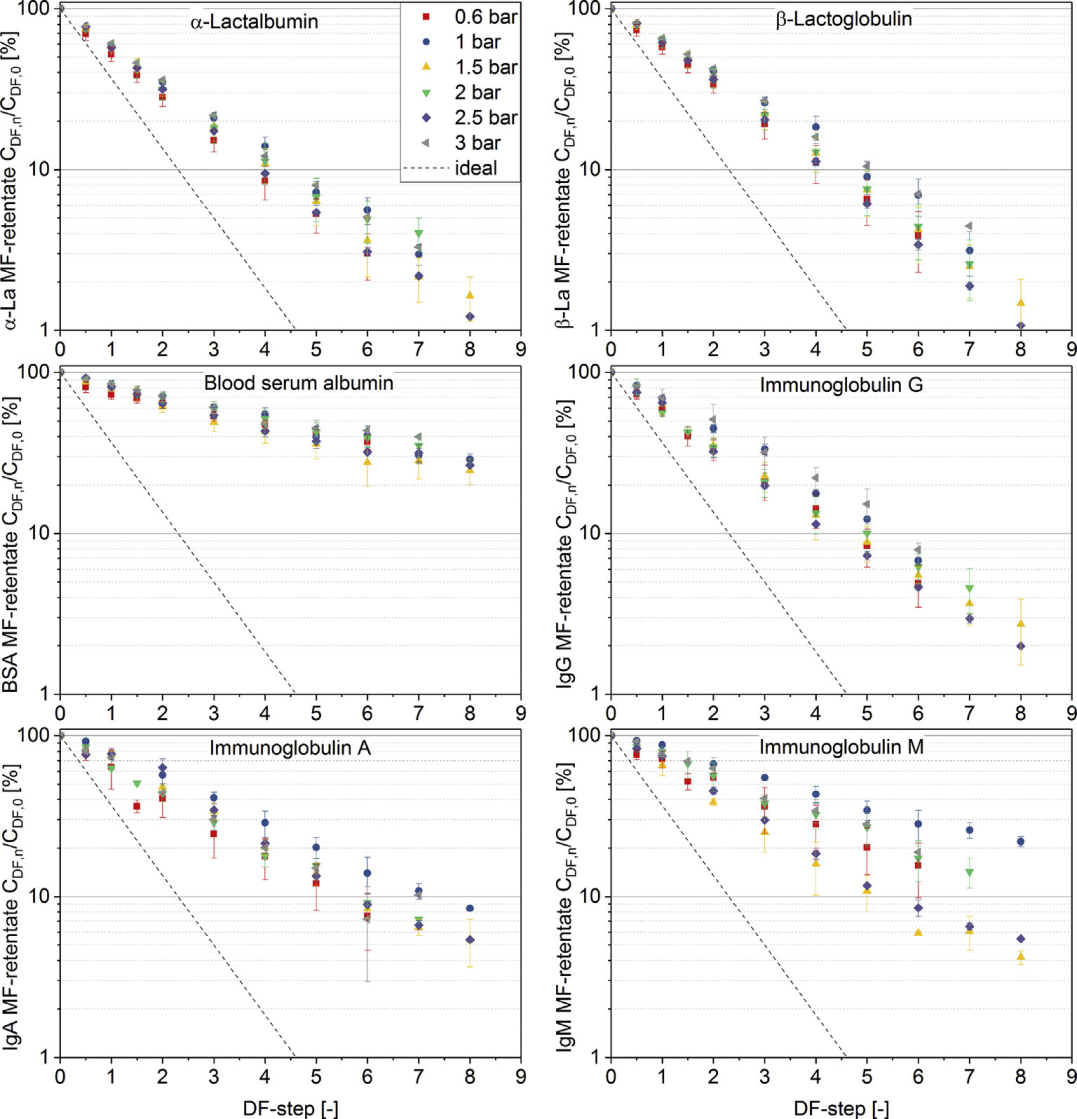
Decrease in the MF-receiving tank of α-La, β-Lg, BSA, IgG, IgA and IgM in comparison to the ideal decrease at 100% transmission and the decrease as a function of the number of DF-steps at indicated TMP (n ≥ 2).

**Fig. 5:**
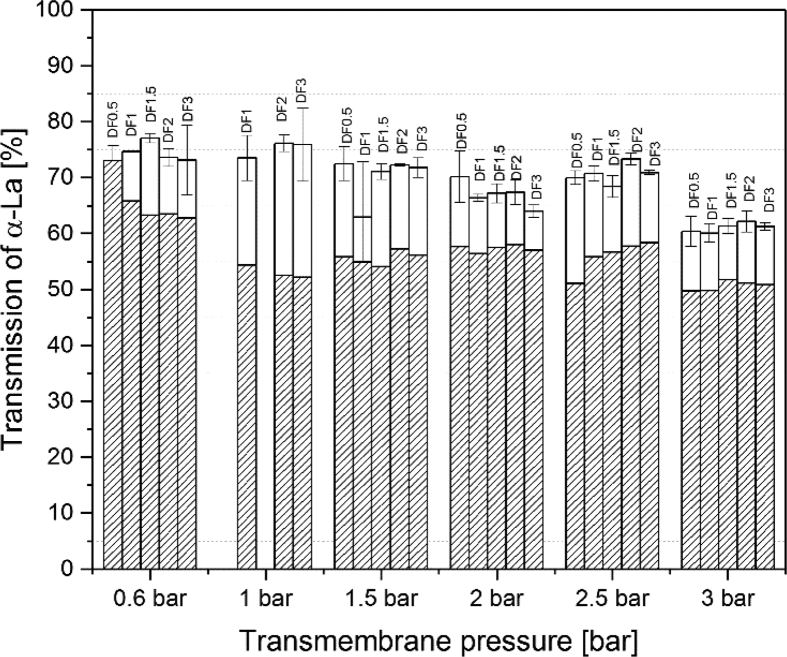
Transmission of α-La determined by Eq. (2) (open bars) and by Eq. (1) (dashed bars). Equations are stated in the related study [1].

**Fig. 6:**
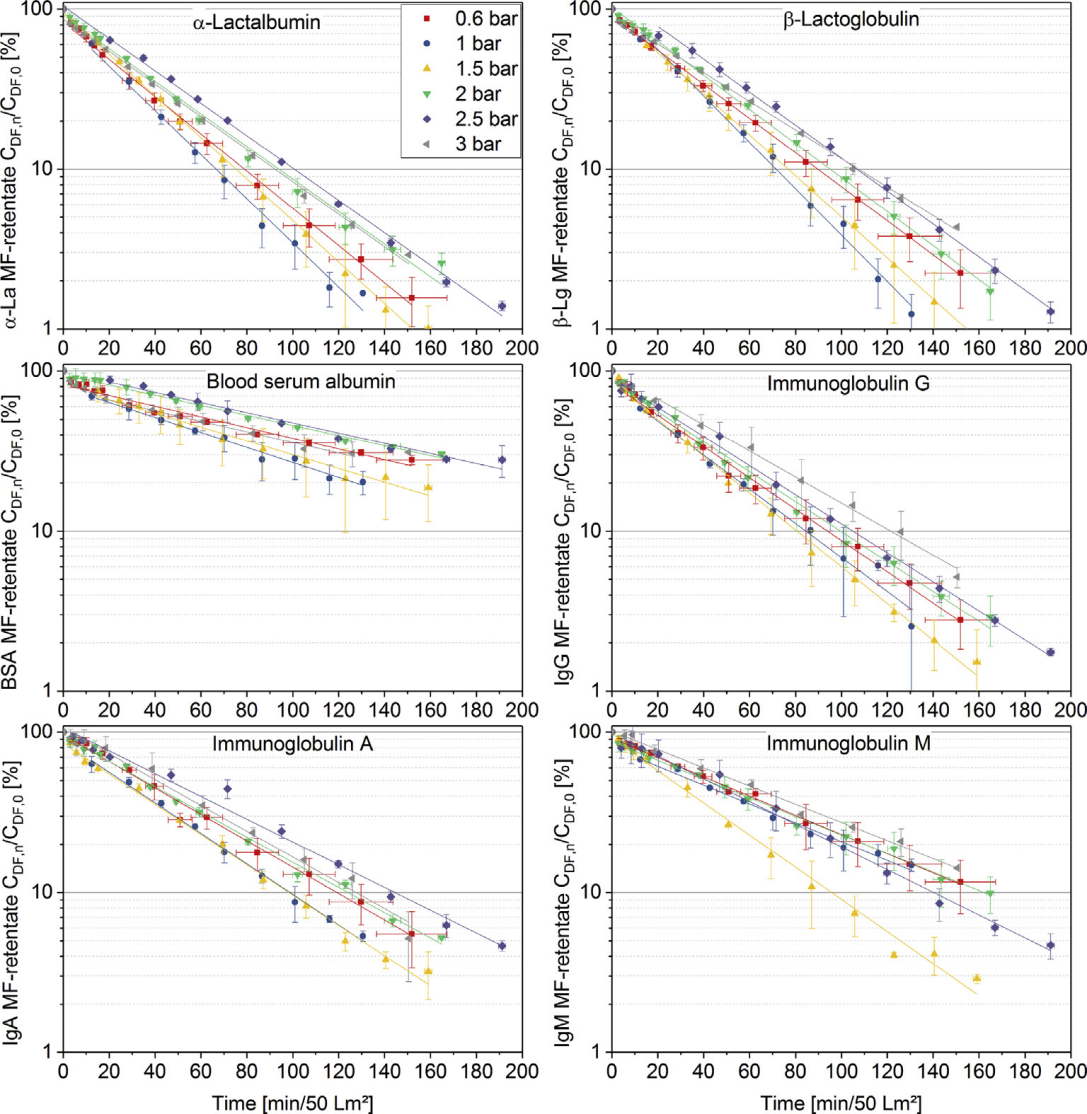
Decrease in the MF-receiving tank of α-La, β-Lg, BSA IgG, IgA and IgM as function of time at TMP of 0.6 bar. The time normalized to 1 m^2^ of membrane area and 50 L of skim milk (n ≥ 2).

**Fig. 7:**
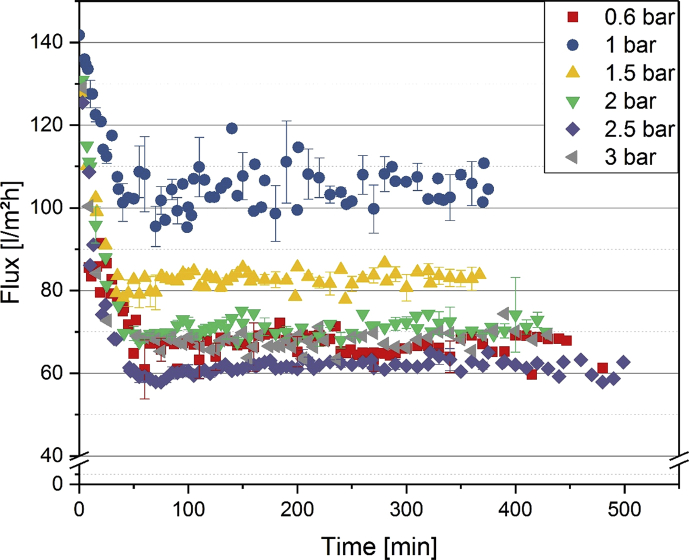
Flux as function of time at indicated TMP (n ≥ 2).

Fig. 1 shows the linear correlation of IgG data measured by ELISA and RR-HPLC. Fig. 2 shows the same correlation for β-Lg measured using two different RP-HPLC methods. Fig. 3 shows the zeta potential as a function of pH and the read of the isoelectric point of IgG and IgM. Fig. 4 shows the depletion of the whey proteins α-La, β-Lg, BSA, IgG, IgA and IgM in the MF retentate as a function of the washing steps. Fig. 5 shows the protein transmission, determined by two different methods using the example of α-La. Fig. 6 shows the time-dependent decrease in the MF retentate and Graph 8 shows the corresponding increase in the UF retentate in the form of a mass balance. Fig. 7 shows the flux at the different transmembrane pressures. Fig. 9 shows the concentration of α-La, β-Lg at 50, 55 °C with respect to the initial concentration in the skim milk during batch filtration and in a water bath. Fig. 10, Fig. 11 show data on the denaturation of β-Lg during filtration operated in diafiltration mode at 50 °C and the selective retention of the insoluble β-Lg by the MF membrane.

## 2 Experimental design, materials, and methods

The preparation of the samples, the equipment and analytical methods for analysis are described in detail by Heidebrecht and Kulozik (2019) [1]. The only method not described was the measurement of the zeta potential, however this methods has been described by Dombrowski et al. (2016) [2].

### 2.1 Comparison of IgG measured with ELISA and RP-HPLC

IgG of different filtration samples were measured with reversed phase high performance liquid chromatography (RP-HPLC) and enzyme-linked immunosorbent assay (ELISA). The binding mechanism to the RP column is based on hydrophobic interactions, while ELISA is used to measure the binding capacity to an antigen.

Fig. 2 shows data on the analytical equivalence of the data generated by ELISA and Reverse Phase High Performance Liquid Chromatography (RP-HPLC), which are two commonly used methods for the detection of IgG. The linear correlation is given by Eq. (1), with (Pearson R^2^ = 0.977) and where y shows the ELISA values and x the HPLC values (Fig. 2).

Eq. (1) y=−0.02718+0.95168·x

For the evaluation of the analytical equivalence, the correlation coefficient according to Lin (1989) was calculated in addition to the stability index according to Pearson [3].

The concordance correlation coefficient was 0.962 with a confident interval of 95% of 0.946–0.974. A coefficient between 0.95 and 0.99 is considered to be an essential correspondence of a set of pairs from two measurements [4]. It should be noted that this correlation may not be valid at high denaturation levels. Native Ig is soluble at pH 4.6 and begins to precipitate at pH below pH 3.5 [5]. Proteins which are not in their native state are precipitated by the adjustment to pH 4.6 and thus not recognized by the RP-HPLC measurement. However, they may have a certain binding capacity and be detected by the ELISA measurement.

### 2.2 Analytical equivalence of two independent RP-HPLC methods for β-Lg quantification

The advantage of the method 1 according to Dumpler et al. (2017) [6] is, that caseins and whey proteins elute one after the other and can therefore be measured in one run. However, IgG cannot be measured with this method, which was the primary goal of the related study. Since caseins and whey proteins do not lie on top of each other, it is possible to determine the total β-Lg content as well as the soluble content at pH 4.6 (also referred to as native β-Lg) and thus to determine the degree of denaturation.

When using the PLRP-S column of method 2 according to Ref. [7], caseins and whey proteins are partly over each other, so that caseins must be precipitated by adjusting the pH to 4.6 during sample preparation. Thus only the native β -Lg content can be measured with this method. The native β-Lg concentration measured with both methods is shown in Fig. 2. The correlation coefficient according to Lin and was 0.995 (confident interval 95% 0.9931–0.9964).

### 2.3 Selective retention of insoluble β-Lg by a 0.14μm filter MF membrane

Fig. 9 shows denaturation data for β-Lg during batch filtration, i.e. directing the permeate back to the retentate. The following data are valuable to determine whether insoluble β-Lg proteins are selectively retained by the MF membrane during filtration in diafiltration mode. Fig. 10 shows the degree of denaturation (DD) defined by Eq. (2) for β-Lg in the MF- and UF-retentate as a function of the filtration time. In Eq. (2) C_A_ is sum of the respective soluble and insoluble whey protein concentration and C_N_ is only the soluble concentration.

Eq. (2) $$DD=\left(1-\;\frac{C_{N}}{C_{A}}\right)\times 100\%\;$$

**Fig. 8:**
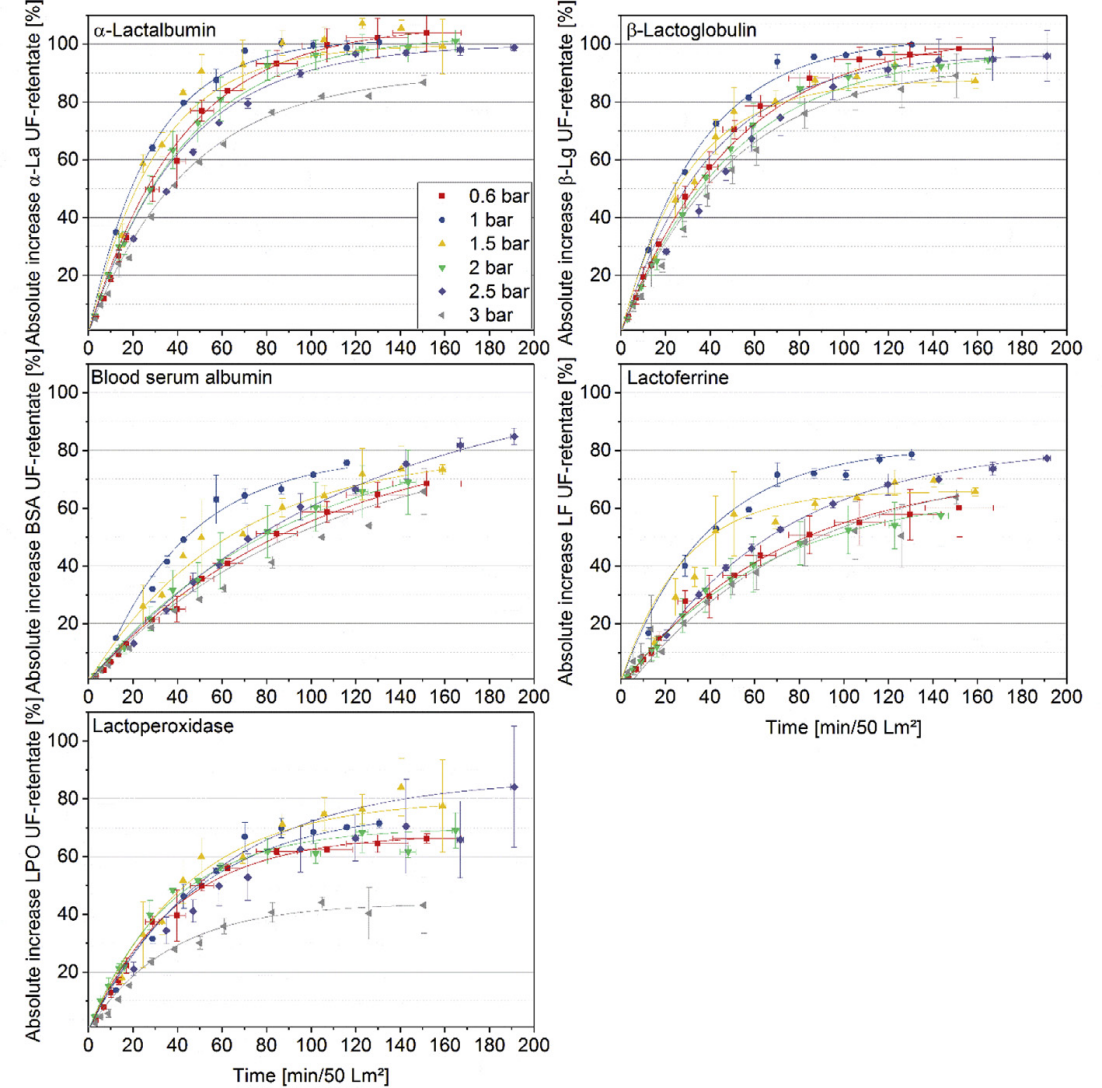
Time dependent mass balance. Measured concentration increase in the UF-receiving tank of α-La, β-Lg, BSA, LPO, LF as a function of time based on 1 m^2^ of membrane area and 50 L of skim milk (n ≥ 2).

**Fig. 9:**
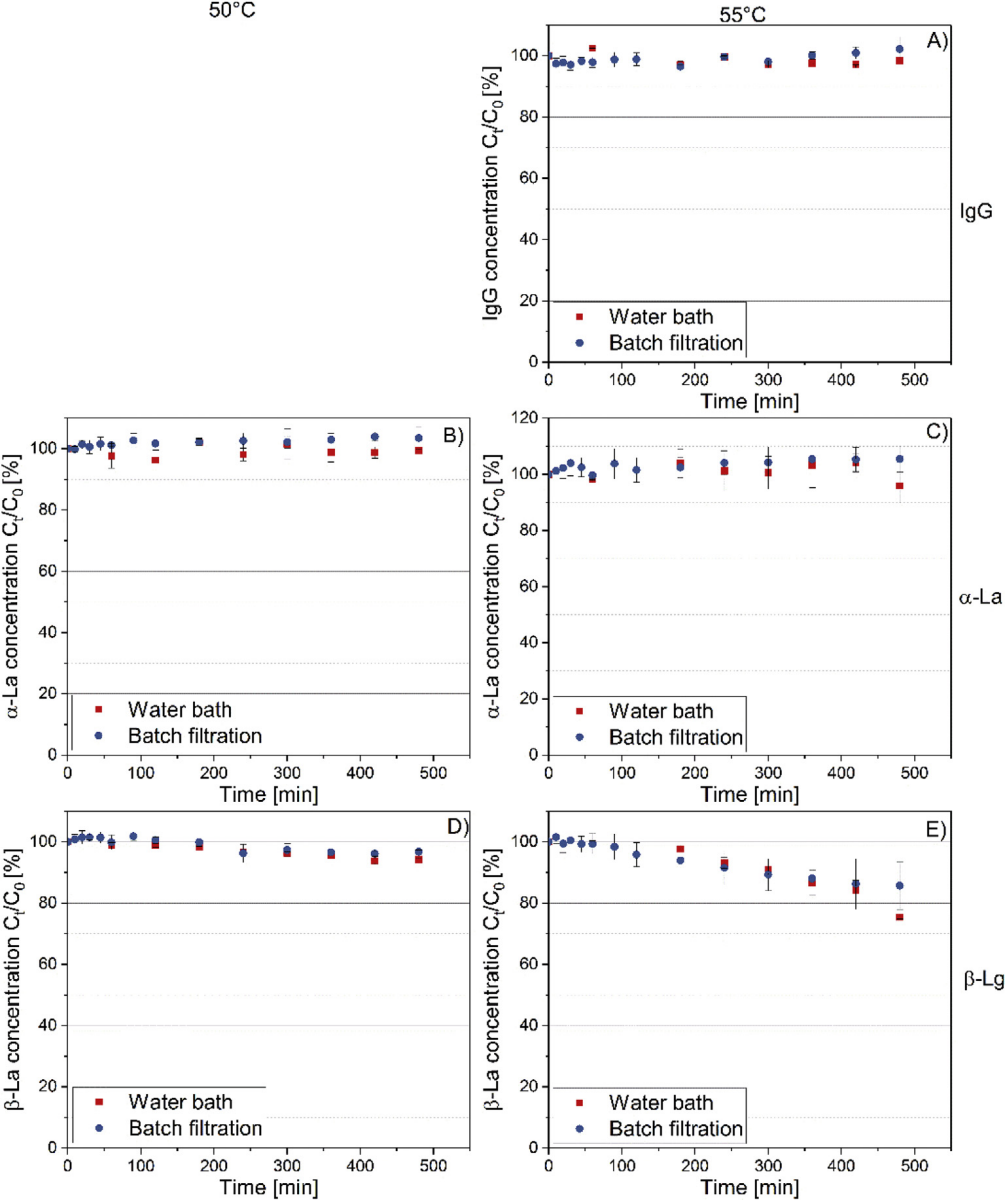
IgG (A), α-La (B,C), β-Lg (D,E) concentration in skim milk during batch filtration at 50C and 55C compared to the respective concentration in a water bath at 50 °C (55 °C) for 8 h. The time-dependent values were related to the intimate concentration in the skim milk (n = 3).

**Fig. 10:**
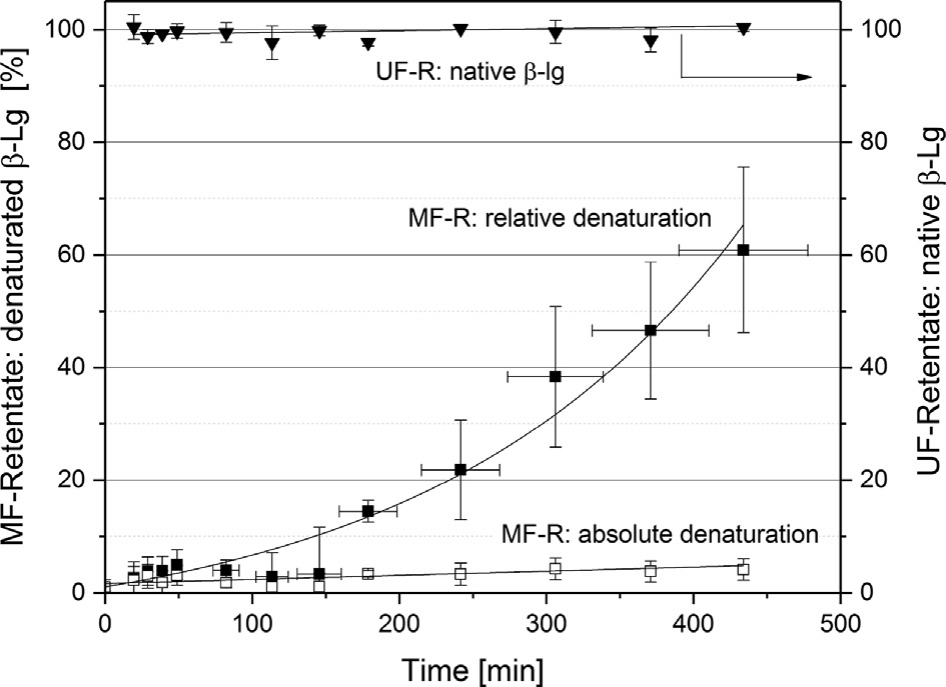
Degree of denaturation (Eq. (2)) and absolute degree of denaturation (Eq. (3)) in the MF-retentate as well as degree of denaturation in the UF feed tank.

**Fig. 11:**
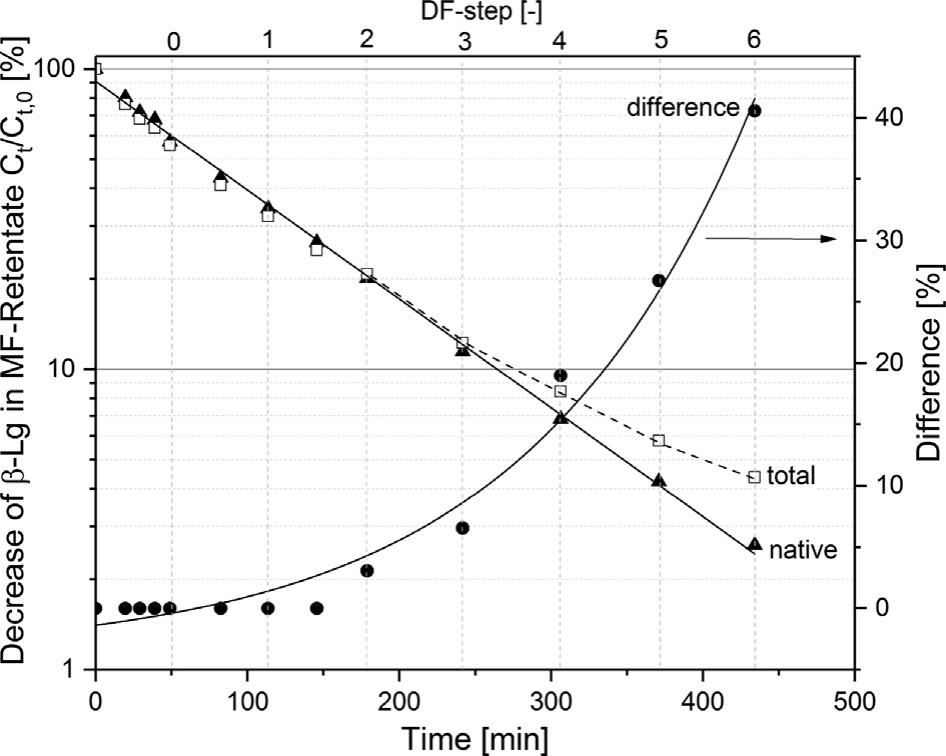
Progress of native and total β-Lg (native plus denatured) as well as the percentage difference of the two curves (right y-axis) as function of the filtration time.

It should be noted that the time scale for Fig. 10, Fig. 11 were not normalized to 1 m^2^ membrane area, as it was the case with Fig. 6, Fig. 8. The DD of β-Lg increased exponentially from 0 to 60% in the MF-retentate, while the data show no denaturation in the UF feed tank. The data in Fig. 10 illustrate the reason for the increase of denatured protein in the MF feed. The concentration of native and total β-Lg in the MF-retentate is the same (the DD is the ratio of these values) at the beginning of the filtration process. The data show that during the filtration process in diafiltration mode native whey proteins pass through the MF membrane, i.e. the concentration of native proteins in the MF-retentate sinks (see Fig. 4, Fig. 6), and the amount of the few aggregated β-Lg continuously increases. At the same time the relative DD in Fig. 11 increases disproportionally. After about 4 DF-steps, the curves deviate from each other, which is better illustrated by the percentage difference in Fig. 11. As the concentration of native proteins decreases, the ratio of native to aggregated proteins in the MF-retentate shifts in the direction of the aggregated (see Fig. 10). However, the 60% denaturation at the end of the DF process is misleading in terms of the nativity of the proteins. Therefore, we also calculated the absolute degree of denaturation (ADD) (Fig. 10), which has the same validity as the DD during batch filtration. The ADD expresses the total amount of denatured protein in the MF-retentate at a given time relative to the total amount of native protein in the milk before filtration. The ADD is defined by Eq. (3) where V_t_ is the volume at a specific time, V_0_ is the initial volume of skimmed milk, C_A_ is the absolute concentration of β-Lg and C_N_ is the native concentration of β-Lg.

(3) $$ADD=\left(\;\frac{V_{t}\cdot \;C_{A\;}-V_{t}\cdot \;C_{N}}{V_{0}\cdot \;C_{N}}\right)\times 100\%$$

The ADD increased to approximately 5% (Fig. 10), confirming 3–4% denaturation during batch filtration (Fig. 9).
